# Expectations of dog and cat owners when dealing with veterinary errors and the emotional impact of such errors

**DOI:** 10.1002/vetr.70226

**Published:** 2026-01-08

**Authors:** Vivian K. Johann, Christin Kleinsorgen, Holger A. Volk, Claudia Busse

**Affiliations:** ^1^ Department of Small Animals Medicine and Surgery University of Veterinary Medicine Hannover Hannover Germany; ^2^ Centre for Teaching University of Veterinary Medicine Hannover Hannover Germany

**Keywords:** communication, critical incident, medical errors, medical error culture, pet owner, veterinarians

## Abstract

**Background:**

This study explored dog and cat owners' experiences of veterinary medical errors and their emotional impact, hypothesising that such errors impose a significant emotional burden.

**Methods:**

German‐speaking dog and cat owners who may have encountered a veterinary medical error were recruited via social media platforms and questioned through an online survey.

**Results:**

A total of 509 pet owners completed the survey, with nearly four out of five owners (78%) reporting to have experienced a veterinary medical error. The predominant emotions associated with errors reported by the affected participants included disappointment (49%), annoyance (48%), sadness (45%) and anger (41%). To help resolve the situation and address the emotional impact, the participants suggested that veterinary surgeons should communicate face‐to‐face with affected owners (84%), be open and acknowledge alleged errors (84%), accept responsibility (73%) and demonstrate commitment to learning and improving following the incident (64%).

**Limitations:**

The social media recruitment process may have introduced bias, favouring those who are present online. In addition, errors were self‐reported by owners and were not confirmed by cross‐checking with medical records.

**Conclusion:**

Medical errors cause a significant emotional burden for pet owners. A proactive, open and transparent approach by veterinary surgeons can mitigate this burden and strengthen the pet‒owner relationship.

## INTRODUCTION

The human‒animal bond is nowadays regarded as being as strong as the relationship with ones’ child.^1^ Additionally, pet owners anticipate treatment and therapy options comparable to those available in human medicine.^2^ To support these expectations, many pet owners turn to the internet to obtain information about their animal's health status.^3^, ^4^ Nevertheless, the internet poses a risk of misinterpretation or misdiagnosis, which can lead to misunderstandings between pet owners and veterinary surgeons.^5^

A previous study showed that the majority of complaints from patient owners addressed veterinary errors,^6^ with the term ‘veterinary error’ not specifically defined. From veterinary surgeons’ perspectives, various types of errors were identified in treatment (71%), parturition (13%), diagnosis (9%), advice (5%) and anaesthesia (2%).^7^ Additionally, a study reports that the most errors are associated with medication (≥54%) and communication (≥21%).^8^

These errors can have significant consequences for patients, their owners and the veterinary surgeons involved, who may experience the ‘second victim effect’.^8^, ^9^, ^10^, ^11^, ^12^ The combination of high‐performance pressure, increasing client expectations and fear of errors are exerting a growing burden on the veterinary profession,^13^, ^14^, ^15^ as veterinary surgeons often worry about negative consequences of errors, including peer judgment and guilt.^10^, ^16^, ^17^ This can lead to psychological problems such as depression, burnout and anxiety, potentially leading to suicide.^18^, ^19^ Nevertheless, the expectations of pet owners when they are confronted with an error remain to be elucidated.

In general, accidents or adverse events can only occur when multiple safety barriers fail. This is illustrated by the Swiss cheese model. Specifically, the slices of cheese represent the safety barriers, which feature small vulnerabilities, indicated by holes in the cheese. If multiple vulnerabilities are connected in a row, there is a risk of an accident or undesirable event.^20^ According to the Swiss cheese model, a serious error does not result from a single cause but is based on several successive minor weaknesses.^21^ The aim of error management should be to identify weak points, intervene in the chain of events at an early stage and thus prevent serious consequences.^22^ Open communication within the team, avoiding victimisation of individuals and working together as a team to find a solution are basic prerequisites for adequate error handling.^23^

The purpose of this study was to investigate the impact of veterinary errors on pet owners, with a focus on understanding the emotional burden from the pet owners’ subjective perspective and factors that may help reducing this burden. Ultimately, this knowledge will help veterinary practitioners to approach and communicate with pet owners in the event of an error and to contribute to the development of a patient‐centered approach.

## METHODS

A cross‐sectional survey of German‐speaking dog and cat owners was conducted using the online software LimeSurvey (LimeSurvey Community Edition version 6.10.3+250203). The data collection period was from January to February 2025.

### Study design

The design of the questions and response options were based on a previous semiqualitative interview study including 23 German pet owners of dogs and cats.^24^ The questionnaire consisted of 25 questions (Appendix 1). These included closed questions, multiple choice questions and semi‐open questions.^25^ Free‐text answers that were possible under ‘other’ were assigned to the already given answers or new categories if necessary. In instances where multiple responses were permitted, a maximum of six answers could be entered simultaneously. The questionnaire contained conditional branching, meaning that not all respondents were asked all the questions equally. The structure of the survey is illustrated in Appendix 1.

### Pretest

The survey was piloted, and five lay individuals checked the comprehensibility of the questions and answer options to test the branching functions and obtain an initial estimate of the time required to complete the questionnaire.^26^

### Development of the study

The participants were asked about their demographic information, frequency of veterinary visits and years of experience as pet owners. The pet owners were asked to share one personal experience of an error, including how it was handled, as well as the consequences it had, and which expectations owners had towards error handling.

### Target population

The inclusion criteria for participation were as follows: participants of legal age, former or current owner of dogs and/or cats, German speaking, and optionally having experienced a veterinary medical error. No prior experience of errors was necessary.

### Recruitment

The link for voluntary participation in the anonymous survey was published via social networks (Instagram, Facebook, *Fehlerkultur Tiermedizin* account), email and direct contacts. The study was advertised with the information that participants were sought who wanted to share their experiences with errors in veterinary medicine. The participants were instructed to focus on a single event that was the most impactful to them.

### Study population

To analyse the survey, the participants were divided into two groups. GroupEr includes respondents who had already experienced an error and participants who may have experienced an error. GroupNoEr represents the participants who had not yet experienced an error, did not know or did not provide any information. The errors were only described from the perspective of the animal owners. It was not evaluated whether the described error was indeed an error from a legal perspective.

### Data analysis

The data were exported as Microsoft Excel spreadsheets and first subjected to descriptive analysis, after which the subsequent statistical analysis was performed in Microsoft Excel. For the evaluation, the participants were divided into two groups. The percentages are rounded to the next whole number and ordinal data were dichotomised.

### Statistical analysis

Patterns and notable findings from the descriptive analysis were tested for statistical significance. Associations between categorical variables were assessed using the chi‐squared test. Univariable logistic regression was performed for binary outcomes, followed where possible by multivariable logistic regression including categorical and continuous predictors. Predictors with small group sizes were excluded from the analysis. Due to multiple responses, multivariable analysis was only done for the type of error, error handling and emotions. The results are reported as odds ratios (ORs) with 95% confidence intervals (CIs); *p*‐values of less than 0.05 were considered significant.

### Data protection

This study was conducted in compliance with the ethical standards of the University of Veterinary Medicine Hannover, Foundation. Data processing was carried out anonymously in line with the university's Data Protection Regulation. No direct identifiers were collected. The institutional ethical and data protection review board approved the study, involving data collection and processing data from humans.

## RESULTS

### Sociodemographic data

A total of 664 individuals accessed the online survey link. After data cleansing and plausibility checks, 509 questionnaires were found to be fully completed (including ‘no‐answer’ options), met the inclusion criteria and were thus deemed suitable for analysis. An overview of the sociodemographic data is provided in Table 1, in which the data have been categorised into groups depending on the experience of an error.

**TABLE 1:** Sociodemographic and pet owner‐related data of included survey participants (*N* = 509).

	GroupEr^a^ (*n* = 397)	GroupNoEr^b^ (*n* = 112)	*p*‐Value	OR	95% CI
Age					
Age^c^ (years) (IQR)	44 (55–35)	48 (55–39)			
≤40 years old	159 (40.1%)	28 (25%)	‒	1	–
>40 years old	216 (54.4%)	75 (67%)	**0.006**	0.507	0.314–0.820
Not specified	22 (5.5%)	9 (8%)			
Gender					
Female	373 (94%)	105 (94%)			
Male	15 (4%)	6 (5%)			
Diverse	3 (0.8%)	–			
Not specified	6 (2%)	1 (0.9%)			
Previous medical knowledge^d^					
Yes	139 (35%)	38 (34%)			
No	245 (62%)	70 (63%)			
Not specified	13 (3%)	4 (4%)			
Experience as pet owner (years) (*n* = 509)					
0–10 years	72 (18%)	34 (30%)	–	1	–
11–20 years	121 (31%)	33 (30%)	0.055	1.731	0.988–3.034
21–30 years	83 (21%)	18 (16%)	**0.019**	2.177	1.134–4.182
>30 years	121 (31%)	27 (24%)	**0.012**	2.116	1.181–3.793
Veterinarian visits on average over the last 6 years (*n* = 501)					
1–2 per year	69 (17%)	39 (35%)	–	1	–
3–4 per year	99 (25%)	31 (28%)	**0.04**	1.805	1.028–3.169
>4 per year	221 (56%)	42 (38%)	**<0.001**	2.974	1.781–4.967
Not at all	1 (0.3%)	–			
Others	4 (1%)	–			
Not specified	3 (0.8%)	–			
Participants' awareness of errors in veterinary medicine					
Yes	98%	96%			
No	1%	2%			
Not specified	1%	2%			

*Note*: Bold indicates statistically significant *p*‐values.

Abbreviations: CI, confidence interval; IQR, interquartile range; OR, odds ratio.

^a^ GroupEr: question ‘Has a veterinary surgeon made an error with your animal?’; answer: ‘yes’; ‘not sure’.

^b^ GroupNoEr: question: ‘Has a veterinary surgeon made an error with your animal?’‘; answer: ’no‘; ’don‘t know’; ‘not specified’.

^c^ Age at the time of evaluation (2025).

^d^ Previous medical knowledge: healthcare professions, for example, nursing, pharmacy, medicine and veterinary assistant.

### Experienced errors in veterinary medicine from pet owners

Out of a total of 509 participants, groupNoEr (*n* = 112) included participants who had never experienced a veterinary error (14%), did not know (7%) or did not specify (0.39%).

The groupEr (*n* = 397) included respondents who had already experienced an error (68%) and those who were not sure if they had experienced an error (10%).

### Types of errors

**FIGURE 1:**
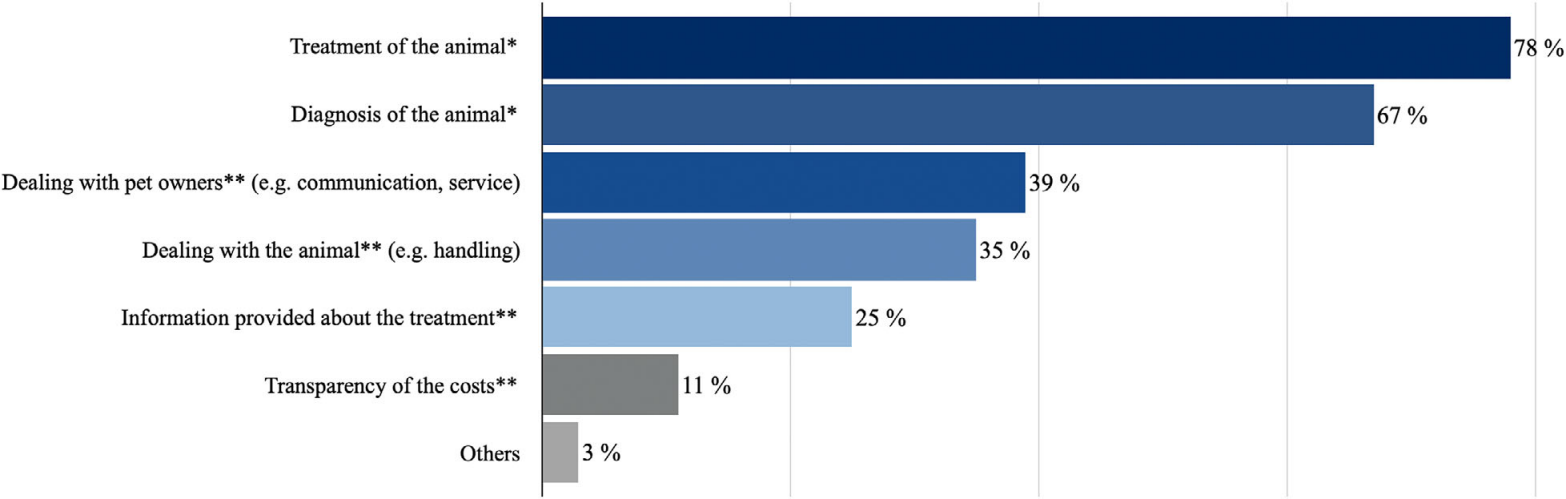
Graphic illustrates the type of errors perceived by pet owners (*n* = 398). Multiple responses possible; ^*^medical errors; ^**^behavioural errors.

According to the participants out of groupEr, the errors reported were medical (42%), behavioural errors of the veterinary team (6%) or both (53%). The most common errors took place in the treatment of an animal (78%). The second most common error occurred in the diagnosis (67%). The remaining aspects in which pet owners identified an error are presented in Figure 1.

Just over half of the participants (55%) noticed the error themselves. The error was noticed by a non‐involved person (29%), by the responsible veterinary surgeon (7%), and sometimes by another person from the same practice (4%). Three percent answered ‘other’ and 1% did not specify.

### Emotions associated with the event

**FIGURE 2:**
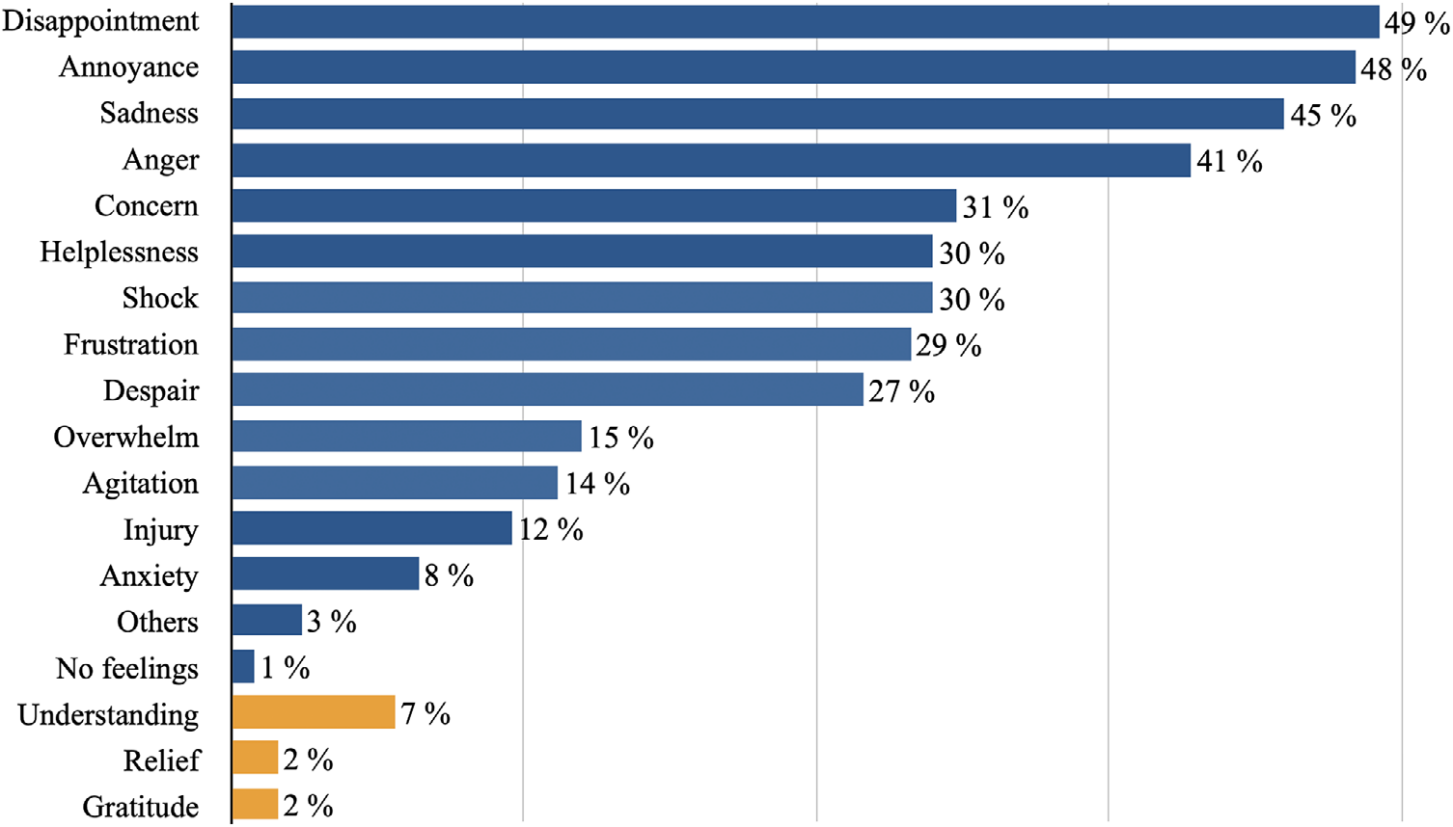
Emotions and reactions of the pet owners in the event of an error; blue: negative emotions, yellow: positive emotions (*n* = 397); multiple responses possible.

The most commonly reported emotional reactions included disappointment (49%), annoyance (48%) and sadness (45%). The most common positive emotional reaction associated with the error was understanding (7%) (Figure 2).

### Perception and conversation about the event

**FIGURE 3:**
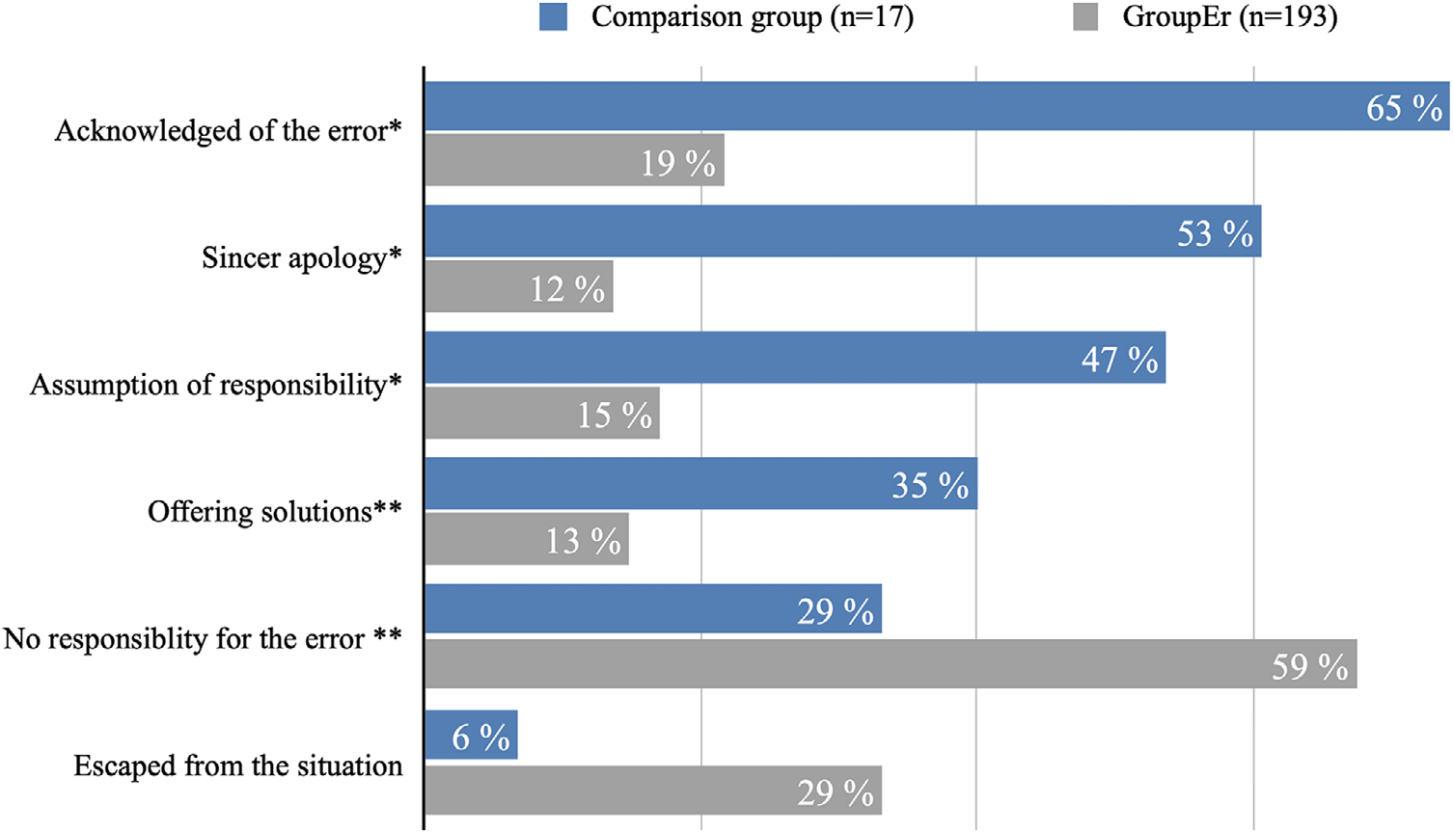
Perceived reactions in a conversation about the error. Participants who felt understood and had a conversation (*n* = 17) compared to the remaining participants from groupEr who had a conversation (*n* = 197); multiple answers possible; ^*^ *p* < 0.001; ^**^ *p* < 0.05.

Fifty‐three percent of the participants had spoken to someone in the veterinary team about the error, while 45% had not and 2% did not specify. Of the 210 participants who had a conversation about the error, 85% spoke with a veterinary surgeon. Over half (56%) of the patient owners had the impression that the person they spoke to did not feel responsible for the error and that the person tried to escape from the situation (27%). Twenty‐three percent of owners felt that the error had been acknowledged, while 16% reported receiving a sincere apology during the conversation. Additionally, 18% perceived that responsibility for the error was accepted, and 15% felt that potential solutions were proposed. An analysis of the participants' responses revealed that those who demonstrated an understanding of the error experienced a significantly more positive conversation than the rest of the groupEr (Figure 3).

The participants who said they had not talked about the error (45%) were asked why they had not done so. Most of the participants felt that a conversation would not have changed the situation (53%). Some participants stated that they were unaware of the situation at the time (33%), while others found themselves overwhelmed (25%) or did not have the courage to say anything (16%).

Of the 178 participants who did not have a conversation about the error, 44% would have liked to talk about the event, while 46% would not and 11% did not specify. Of the participants who would have liked to talk about the incident, 68% would have liked to speak with the responsible veterinary surgeons, 37% with an uninvolved person and 17% would have liked to talk to the supervisor of the responsible person.

### Consequences due to the event

Trust in the responsible veterinary surgeons was lost for 44% of the participants from groupEr and had been reduced for 37% of the participants in groupEr because of the event. Trust remained unchanged for 14% of patient owners and even improved for 2%. Sixty‐two percent of the participants said they would not return to this veterinary practice under normal circumstances (e.g., for a routine check‐up), while 34% said they would and 5% did not specify.

The experienced error had negative consequences for 80% of participants, while 17% reported that the error had no consequences and 4% did not specify. The most commonly reported consequence was an increased suffering for the animal (81%). Emotional distress for the pet owner (54%), increased costs (51%) and increased care requirements for the animal (31%) were also listed. Forty‐five percent of the participants changed their veterinary practice as a result of the error. Furthermore, 16% of pet owners lost their pet through death or euthanasia.

### Comparative analysis

#### Sociodemographic data

Within the groupEr, it was found that individuals who were uncertain were significantly less likely to experience an error in the medical and behaviour‐related field (compared to medical errors; OR = 0.441, 95% CI: 0.231‒0.842, *p* = 0.013). Furthermore, these individuals were significantly less likely to report prior medical knowledge (compared to those with no prior medical knowledge; OR = 0.224, 95% CI: 0.093‒0.543, *p* < 0.001).

#### Factors influencing emotions

It is noteworthy that among the most frequently cited emotions, participants who were annoyed were 2.5 times more likely to believe that the error was medically and behaviourally related (vs. medical errors; OR = 2.476, 95% CI: 1.611‒3.806, *p* < 0.001). In addition, participants who were angry were 1.8 times more likely to believe that the error was of a medical and behavioural nature (vs. medical errors; OR = 1.760, 95% CI: 1.138‒2.722, *p* = 0.011).

The participants who experienced consequences of errors had higher odds of reporting anger (OR = 3.3914, 95% CI: 2.016‒7.599, *p* < 0.001) and sadness (OR = 1.762, 95% CI: 1.009‒3.007, *p* = 0.046) compared to those without consequences. Moreover, divergent strategies for dealing with errors were evident, depending on the emotions involved. The participants who expressed annoyance were more likely to receive an apology than those who did not express such an emotion (OR = 1.565, 95% CI: 1.028‒2.382, *p* = 0.037).

In the context of disappointment, there was a clear preference for assuming responsibility (OR = 1.935, 95% CI: 1.228‒3.48, *p* = 0.004) and offering solutions (OR = 1.592, 95% CI: 1.065‒2.380, *p* = 0.023). The emotion of anger led to a significantly stronger desire for an apology (OR = 1.771, 95% CI: 1.151‒2.25, *p* = 0.009), for the costs to be covered (OR = 1.871, 95% CI: 1.239‒2.825, *p* = 0.003) and for the ability to express displeasure (OR = 4.675, 95% CI: 1.195‒18.294, *p* = 0.027). However, the parameter ‘expressing displeasure’ should be interpreted with caution due to the small sample size.

#### Factors influencing the preferred handling of errors

A significant correlation was identified between the desire to learn from errors and the type of error. Individuals who had experienced an error in behaviour were significantly less interested in the veterinary team learning from errors than individuals who had experienced a medical error (OR = 0.406, 95% CI: 0.168‒0.983, *p* = 0.046).

Individuals who had engaged in a prior conversation had increased odds of wishing to communicate with the veterinary team (OR = 1.840, 95% CI: 1.072‒3.157, *p* = 0.027) and a coverage of the costs (OR = 1.529, 95% CI: 1.022‒2.290, *p* = 0.039). Conversely, the participants who had a prior conversation less often expressed a wish for understanding from the veterinary team when dealing with errors (OR = 0.55, 95% CI: 0.341‒0.888, *p* = 0.014).

Overall, both groups had similar preferences with regard to the management of errors.

When comparing the groupEr and groupNoEr, it was evident that open communication with those affected (OR = 2.146, 95% CI: 1.066‒4.318, *p* = 0.032), openness to recognising and acknowledging errors (OR = 4.183, 95% CI: 1.771‒9.881, *p* = 0.01), and proposing solutions (OR = 1.699, 95% CI: 1.093‒2.639, *p* = 0.018) were significantly more frequently desired by groupNoEr. In contrast, the desire for the veterinary team to learn from errors in order to avoid them in the future was significantly less prevalent among groupNoEr (OR = 0.603, 95% CI: 0.393‒0.923, *p* = 0.02) (Table 2).

**TABLE 2:** Desired handling of errors from the participants' perspective.

	GroupEr	GroupNoEr	*p*‐Value	OR	95% CI
Desired handling of an error					
Communication with those affected (i.e., pet owner)	328 (83%)	102 (91%)	**0.032** ^a^	2.146	1.066–4.318
Open to recognise and acknowledge an error	321 (81%)	106 (95%)	**0.01** ^a^	4.183	1.771–9.881
Responsibility for the error	288 (73%)	83 (74%)	0.742^a^	1.083	0.672–1.45
Learning and development of the veterinary team to avoid errors	264 (67%)	61 (54%)	**0.02** ^a^	0.603	0.393–0.923
Being offered solutions	216 (54%)	75 (67%)	**0.018** ^a^	1.699	1.093–2.639
Coverage of costs for damage incurred/incorrect treatment	185 (47%)	61 (54%)	0.142^a^	1.371	0.900–2.088
Apology	132 (33%)	34 (30%)	0.64^a^	0.875	0.556–1.377
Understanding of feelings by the veterinary surgeon	92 (23%)	28 (25%)	0.688^a^	1.105	0.679–1.799
Emotional support from the veterinary team	61 (15%)	18 (16%)	0.855^a^	1.055	0.595–1.871
Emotional support from friends/relatives	12 (3%)	3 (3%)	0.849^a^	0.883	0.245–3.185
Express your displeasure (e.g., through online reviews, at the registration desk, etc.)	11 (3%)	1 (1%)	0.273^a^	0.316	0.040–2.475
Others	8 (2%)	1 (1%)			
Error reporting system					
Yes	266 (67%)	51 (46%)	**<0.01** ^b^		
No	84 (21%)	44 (39%)			
Not specified	47 (12%)	17 (15%)			

*Note*: Bold indicates statistically significant *p*‐values. GroupEr: *n* = 397 and groupNoEr: *n* = 112. Multiple answers are possible.

Abbreviations: CI, confidence interval; OR, odds ratio.

^a^ Binary logistic regression.

^b^ Chi‐squared test.

In the context of the investigation into the necessity for an error reporting system, it is significant that individuals who had previously experienced an error were more likely to want an error reporting system (67%) than those who had not (46%) (*p* < 0.01, chi‐squared test).

Participants aged over 40 years had significantly lower odds of desiring openness (OR = 0.543, 95% CI: 0.316‒0.932, *p* = 0.027) and solution‐oriented approaches (OR = 0.685, 95% CI: 0.471‒0.995, *p* = 0.047) compared to those aged 40 years or under. Conversely, participants aged over 40 years more frequently wished for getting the costs covered (OR = 1.541, 95% CI: 1.063‒2.223, *p* = 0.022).

Participants with 11–20 years of pet ownership experience had higher odds of desiring responsibility‐taking compared to participants with 10 years or less of experience (OR = 1.972, 95% CI: 1.138‒3.419, *p* = 0.016), as did those with 21–30 years of experience (OR = 2.007, 95% CI: 1.083‒3.720, *p* = 0.027). Those with over 30 years of experience more often wished for proposed solutions (OR = 0.475, 95% CI: 0.285‒0.790, *p* = 0.004).

## DISCUSSION

The conducted questionnaire‐based study showed that medical errors in veterinary medicine cause emotional distress for pet owners. Those who have experienced an error commonly report feelings of disappointment, frustration, sadness or anger. Beyond their own emotional burden (54%), the majority perceived that the error led to increased suffering of their animal (81%) and increased costs (51%). In response to errors, pet owners strongly prefer open communication from the veterinary surgeon (84%). Owners wanted the veterinary surgeon to take responsibility for the situation (73%) and for the error to be acknowledged and recognised (84%). Additionally, 62% of the participants expressed support for an anonymous reporting system for errors and near misses.

### Types of errors

A previous study conducted a retrospective case analysis and categorised errors in treatment (surgical and medical) (71%), parturition (surgical, medical) (13%), diagnosis (9%), advice (5%) and anaesthesia (2%) claims.^7^ In our study, the majority of owners identified errors in the treatment of the animal (78%) and in the diagnostic process (68%). These types of errors can be classified as medical errors. The perception of the frequency of errors in ‘treatment’ from the perspective of veterinary surgeons^7^ and animal owners in our study is similar. However, there is a large discrepancy between the perception of errors in diagnosis in our study (68%) and the findings in retrospectively analysed cases of diagnostic errors (9%). Nevertheless, in the present study, different types of errors were selected by the patient owners, according to their own assessment. It is a subjective perception from the patient owners’ point of view, which cannot be analysed further in this study, and owners are also unlikely to discover errors in other less accessible aspects, such as anaesthesia or structural processes.

In our study, 39% of pet owners noticed an error occurring in interactions with them. This suggests that while communication within the veterinary team can lead to errors, communication with pet owners should also be considered a significant source of error. Other studies have shown that communication issues between veterinary surgeons and pet owners are more likely to result in complaints.^6^, ^27^, ^28^ One study revealed that in 80% of cases involving alleged veterinary negligence, communication problems were identified as contributing factors.^28^ A substantial portion of these errors occurred during direct interactions with pet owners. These communication challenges occured at the levels of context, content and perspective They were characterised by situations in which pet owners felt unheard and where not involved in a shared decision‐making process.

In the present study, many participants felt that the person did not accept responsibility for the error (56%) and tried to escape from the situation (28%). However, the perceived behaviour can only be an assumption, as we only collected data from the perspective of the patients' owners, which is likely to be affected by a recall bias. One potential incentive for the responsible parties to disclose the error could be that individuals often experience a sense of relief through the expression of their emotions.^29^ Furthermore, veterinary surgeons face a dilemma between the desire to apologise and the fear of legal consequences.^30^ It was found that these factors not only delayed disclosure but also contributed to emotional stress and moral injury on the part of the veterinary surgeons.

In order to minimise the source of error in communication with pet owners, an improved communication structure should therefore be established to communicate errors transparently in the future.

### Handling of errors

A survey in human medicine found that patients wanted to be informed about an error if it has caused them harm.^31^ Patients expected a basic description of what the error was, why it occurred and how it will be prevented in the future. An apology was also an important aspect to mitigate their emotional experiences following an error.^31^ In our study, the patient owners expressed a preference for open communication and transparency by acknowledging the error. These preferences have also been reported in another study that looked at the experiences and expectations of pet owners when dealing with errors in veterinary medicine.^32^ Additionally, our study found that individuals who felt understood exhibited a significantly more positive outcome when discussing the error (Figure 3). Conversely, positive emotions have been demonstrated to be associated with positive conversations.

A recent study in human medicine has demonstrated that trust between patients and their doctors can be supported through open communication in the event of an error.^31^ In addition, in veterinary medicine, the practice of sharing adverse events and near misses with customers has also been shown to increase the satisfaction of veterinarians themselves.^10^

It is evident that the quality of the conversation can influence pet owners' perceptions of an error. Pet owners expected honest and empathetic communication from the veterinary surgeon, in which their concerns are taken seriously, and their decisions about the animal treatments are respected.^27^, ^33^, ^34^, ^35^, ^36^, ^37^ Honesty, compassion for people and animals and good communication skills were essential in everyday practice work, as well as in critical situations.^38^, ^39^

### Reporting system

A reporting system for veterinary professionals can have the benefits such as receiving feedback, protection against disciplinary action for reported errors, opportunities for shared learning and structured reporting processes.^40^ However, within the veterinary team, concerns have been raised about the potential misuse of error reports, along with challenges related to time constraints and organisational logistics.^40^

Notably, examples from the UK illustrate how veterinary error reporting systems for veterinary professionals can improve understanding of error types, causes and patterns. These systems enable learning from incidents, system improvements, enhanced patient safety and the promotion of a positive culture around error reporting in veterinary medicine.^41^, ^42^

In addition, the benefits of a reporting system for patients in human medicine have been proven. With patients’ help, errors can be detected and patient safety increased using this information.^43^, ^44^ An internal reporting system within the clinic also allows connections to be made to reported cases, which can then be dealt with appropriately. Our findings demonstrated that pet owners who had previously experienced an error were also more likely to be in favour of an error reporting system compared to those who had not, indicating a heightened interest in the subject.

Similarly, an accessible error reporting system for animal owners could facilitate recognition and learning from errors, ultimately enhancing patient safety in veterinary practice. It is imperative to consider potential regional variations; within Europe, there is already evidence that reporting behaviour exhibits disparities among different countries.^9^ The role of cultural, sociological and other influences on reporting behaviour, as well as their potential impact on the handling of errors, constitutes a promising avenue for further investigations.

### Limitations

A notable limitation of the present study was the reliance on data from pet owners alone, which provided a subjective and one‐sided view of the situation.^29^ Furthermore, the participants’ responses were influenced by pre‐established answer options. The survey may be influenced by order effects, whereby the participants’ answers were influenced by the questions asked previously and by self‐reporting bias, depending on their motivation, reading skills, level of information and frame of reference of the participant to the survey topic.^45^ It can be assumed that recall bias was also present, as the data collected were based on the animal owners' memories.^46^, ^47^

A higher proportion of participants who had encountered an error (78%) answered our questionnaire. This is likely due to the fact that people who have a direct connection with the research topic are more likely to take part in the survey.^48^ The recruitment process took place through social media, a practice that has the potential to introduce bias, resulting in a sample population mainly comprised of individuals who are active on these platforms.

The study was limited by technical constraints, which made it difficult to collect data on multiple events. As a result, participants were asked to report on only one event, which represents a limitation as it restricts the findings to a single experience per participant.

The statistical tests used also have several limitations. The chi‐squared test can be unreliable with small cell counts (<5), and univariable analyses do not account for potential confounders. Multivariable logistic regression requires sufficient events per variable and can be biased by multicollinearity; moreover, it was only applicable for certain outcomes due to multiple responses. Importantly, the reported ORs reflect associations and should not be interpreted as causal effects.

In the context of further research, it would be beneficial to solicit the perspectives of veterinary surgeons on their error management practices to determine whether veterinary surgeons’ expectations align with those of pet owners. In the event of an error, it would be beneficial to interview both veterinary surgeons and pet owners in order to consider the views of both parties. The piloting and evaluation of acceptance and effectiveness of an error reporting system for pet owners could also be of interest.

## CONCLUSION

The emotional reactions that occur in situations involving errors in veterinary practice remain an important area for research. We could show that errors in veterinary medicine result in emotional distress for the owners. The most common emotions associated with errors in veterinary medicine were disappointment, annoyance, sadness and anger. It has shown that especially individuals who have previously experienced an error wish for a transparent and honest discussion about the error, that the error is recognised and acknowledged and that those responsible take responsibility for it, ideally ensuring that the same mistake will not occur again.

## AUTHOR CONTRIBUTIONS

Vivian K. Johann designed and conducted the study on errors in veterinary medicine from the perspective of patient owners. Christin Kleinsorgen, Holger A. Volk and Claudia Busse contributed to the design of the study. Vivian K. Johann performed the data collection and statistical analysis and wrote the first draft of the manuscript. All the authors contributed to manuscript revision and approved the final version for submission.

## CONFLICT OF INTEREST STATEMENT

The authors declare that the research was conducted in the absence of any commercial or financial relationships that could be construed as a potential conflict of interest.

## ETHICS STATEMENT

The institutional review board of the university approved the project in accordance with ethical guidelines for research involving human participants. Furthermore, the university's Data Protection Officer granted approval for the project. All participants voluntarily consented to the processing of their data in accordance with the EU General Data Protection Regulation (2018), specifically Article 6(1)(e) in conjunction with Article 89, as well as the Lower Saxony Data Protection Act (§ 3(1) No. 1 NHG, § 13).

## Supporting information



Supporting Information

## Data Availability

All data are included in this published article and its Supporting Information files.
