# Supplementary material for: Expectations of dog and cat owners when dealing with veterinary errors and the emotional impact of such errors
Source: Vet Rec. 2026 Jan 8;199(3):e106–14. doi: 10.1002/vetr.70226 (PMC13425710; doi:10.1002/vetr.70226)
Supplement: Supplementary file 1 — Supporting Information [file VETR-199--s001.pdf]

In which year were you born?

- Free text

↓

What is your sense of gender identity? (SC*)

- Female | Male | Diverse
- Other: free text
- Not specified

↓

Do you have any previous medical knowledge? (SC)

- Yes | No | Not specified

↓

Over the past six years (2018-2024), how many times a year, on average, have you visited a veterinary practice with your pet(s)? (SC)

- Not at all
- 1-2 per year
- 3-4 per year
- More than 4 times a year
- Other: free text
- Not specified

↓

Since how many years have you been a pet owner? (SC)

- 0-10 years
- 11-20 years
- 21-30 years
- Over 30 years
- I'm not a pet owner
- Other: free text
- Not specified

Are you aware that errors do happen in veterinary medicine? (SC)

- Yes | No | Not specified

Has a veterinary made an error with your animal? (SC)

Yes

How many errors have you experienced in the veterinary care of your animal(s) in the last 10 years (2014 - 2024)? (SC)

- 1 | 2 | 3 | 4 | 5
- More than 5
- Not specified

Not sure

No
Didn't know
Not specified

In your opinion, what would have been the error? (MC**)

- In dealing with your pet (handling, appreciation as a family member, etc.)
- In dealing with you (as a patient owner) (e.g. communication, service, etc.)
- Treatment / therapy of your animal
- Information provided about the treatment
- Transparency of costs
- Diagnosis / diagnostics of your animal
- Other: Free text

In which species of animal has an error been made? (SC)

- Dog | Cat | Not specified

How did you find out about the error? (SC)

- Other persons from the responsible veterinary practice
- The attending veterinary surgeon has told you about the error
- An uninvolved third party has pointed this out to you (e.g. another veterinary practice, friends, acquaintances, etc.)
- You noticed the error yourself
- Other: Free text

What feelings do you have about this event? (MC)

- No feelings
- Annoyance
- Disappointment
- Frustration
- Sadness
- Anger
- Injury
- Anxiety
- Despair
- Overwhelm
- Concern
- Shock
- Agitation
- Helplessness
- Gratitude
- Relief
- Understanding
- Other: free text

Have you spoken to anyone in the veterinary team about the error? (SC)

Yes

Whom did you have a conversation with about the error? (MC)

- Person on the phone
- Person at the registration desk
- Veterinary surgeon
- Veterinary assistant
- Other: Free text
- Not specified

No

Not specified

Why do you didn't want to talk about the error? (MC)

- A conversation wouldn't have changed the situation.
- I didn't have the courage to say anything.
- I was overwhelmed in the situation.
- At the time, I didn't realise that something had gone wrong.
- Other: free text

How did the person respond to your concern? (MC)

- The error was acknowledged
- A genuine apology was offered
- Responsibility for the error was accepted
- Solutions were suggested
- No sense of responsibility was felt for the error
- An attempt was made to flee the situation
- Other: Free text

Would you like to talk about the event now? (SC)

No
Not specified

Yes

Who would you want to talk to about this? (MC)

- With the person responsible
- Supervisor of the person responsible
- An uninvolved person (e.g. other vets, friends, family, etc.)
- Others: free text

How has trust in the responsible veterinarians changed as a result of the error? (SC)

- Remained the same
- Deteriorated
- Improved
- No more trust
- Other: Free text
- Not specified

Would you return to these veterinary surgeons under normal circumstances, e.g. for a routine examination (not an emergency situation)? (SC)

- Yes | No | Not specified

Did the error have consequences for you or your animal? (SC)

Yes

No
Not specified

Which consequences had the error? (MC)

- Increased costs
- Increased care requirements
- Emotional stress
- Increased suffering for your pet
- Change of veterinary practice
- Other: free text

What is important to you when an error has happened to your animal? (MC)

- Open, to recognise and acknowledge an error
- Responsibility for the error
- Communication with those affected (i.e. pet owners)
- Apology
- Being offered solutions
- Coverage of costs for damage incurred / incorrect treatment
- Emotional support from the veterinary team
- Emotional support from friends / relatives
- Understanding of feelings by the veterinary surgeon
- Learning and development of the veterinary team to avoid errors
- Express your displeasure (e.g. through online reviews, at the registration desk, etc.)
- Other: free text

If your veterinary surgeon made an error when treating your pet, what would be most important to you? (MC)

- Open, to recognise and acknowledge an error
- Responsibility for the error
- Communication with those affected (i.e. pet owners)
- Apology
- Being offered solutions
- Coverage of costs for damage incurred / incorrect treatment
- Emotional support from the veterinary team
- Emotional support from friends / relatives
- Understanding of feelings by the veterinary surgeon
- Learning and development of the veterinary team to avoid errors
- Express your displeasure (e.g. through online reviews, at the registration desk, etc.)
- Other: free text

Would you like a reporting system (e.g. an anonymous online platform) where you can report errors or near-misses in veterinary medicine? (SC)

- Yes | No | Not specified

*SC: Single Choice
** MC: Multiple Choice (max. 6 answers)
